# Role of Food Digestion and Digestive System in the Nutritional, Functional and Health Properties of Food Bioactives

**DOI:** 10.3390/nu16050712

**Published:** 2024-02-29

**Authors:** Samuel Fernández-Tomé

**Affiliations:** Department of Nutrition and Food Science, Faculty of Pharmacy, Complutense University of Madrid (UCM), Plaza Ramón y Cajal s/n, 28040 Madrid, Spain; sfernandeztome@ucm.es

The impact of food components on the human digestive system is an important area of research in the fields of nutrition and food science. Food is composed of a mix of chemical components that will be processed by the digestive system to release, absorb, and metabolize the nutrients. The composition and structure of food play a significant role in its nutritional and functional properties, making it necessary to understand the modifications and mechanisms occurring during food digestion to better comprehend the potential health benefits of food bioactives. Furthermore, the human gastrointestinal tract is continually exposed to a varied range of food compounds in the gut lumen as they are processed following oral intake and then transported across the intestinal barrier. Food compounds may undergo several changes in the digestive system; hence, it is a real challenge for food scientists to understand their health implications within and beyond the gastrointestinal tract. Researchers have thus utilized some in vitro models in an attempt to simulate the gastrointestinal digestion process and study the interaction between foods and the digestive system, but the complexity of this interface require further investigation to advance our understanding of food digestion, bioaccessibility, and bioavailability, along with the influence of gut microbiota. The field of functional foods aims to uncover these complexities on the path towards enhancing our knowledge on the impact of food bioactives on human health and disease.

This Special Issue of the journal *Nutrients* includes eight outstanding papers describing examples of the new research trends on the interplay between the following key elements in nutrition and food science research: (i) the physiology of the digestive system and gastrointestinal digestion of foods; (ii) the analysis of the food matrix and composition during digestion; (iii) the nutritional, functional, and biological properties of food bioactives; and (iv) the health impact of the modulation of digestive functions through dietary substances. 

The Special Issue begins with a group of research articles investigating different aspects related to the digestibility of food proteins (Contributions 1–4). The study of Benede et al. (Contribution 1) aimed at evaluating the susceptibility to digestion of transglutaminase-mediated crosslinked skim milk and its two main protein fractions, namely casein and whey protein, as well as their allergenic potential. After treatment with transglutaminase, the human IgE binding capacity of sera from milk-allergic patients to whey protein was increased but reduced in the case of skim milk and casein. Despite no effects being provoked in mast cell degranulation, in comparison to native proteins, the cross-linking of skim milk and casein induced a lower release of Th2 cytokines from the splenocytes of sensitized mice. Moreover, all transglutaminase-treated milk samples were more resistant to simulated digestion, and, consequently, the binding to human IgE of their digests was increased. This fact questioned the use of transglutaminase-crosslinking to obtain hypoallergenic products for cow’s milk allergic patients and highlighted the necessity to evaluate the whole food matrix composed of diverse compounds with different resistance to processing and gastrointestinal digestion and, therefore, showing different interactions with gut mucosa and allergenicity mediators [1,2].

Nowadays, it is a well-known fact that the COST Action INFOGEST was established as an international network with the final aim of “Improving health properties of food by sharing our knowledge on the digestive process”. It has been working on food digestion for several years and currently gathers more than 440 researchers from more than 40 countries worldwide. Hence, the network reached a consensus to harmonize the in vitro static systems that simulate the digestive processes by defining key parameters and conditions, hence allowing the publication of the standardized static in vitro digestion method suitable for food developed by Minekus et al. [3]. Noteworthy, the methodology achieved an extraordinary impact in the scientific community, currently reaching over 3300 citations since its origin from multiple research institutions worldwide, and it has been amended in an updated publication of the improved digestion protocol (INFOGEST 2.0) [4]. In this Special Issue, the study of Correa et al. (Contribution 2) applied this simulated digestion method to evaluate the release of essential amino acids and bioactive peptides from the legume *Erythrina edulis (chachafruto)* proteins during gastrointestinal digestion. Legume-derived food peptides have been shown in the literature to display mostly antihypertensive, antimicrobial, antidiabetic, or antiproliferative properties [5]. In this specific study on chachafruto proteins, low molecular weight peptides released after protein digestion were proved to exert antioxidant activity through radical scavenging capacities, whereas high molecular weight peptides prompted both immune activation in RAW 264.7 macrophages and cell protection against LPS challenge.

In the study of Jiménez-Muñoz et al. (Contribution 3), a semi-dynamic INFOGEST-based digestion system provided useful information about optimal gastrointestinal conditions for protein bioaccessibility, but also about the mechanistic understanding of how food matrix may influence the behavior of proteins during gastric and intestinal digestion. Hence, the study made use of confocal laser scanning microscopy, and protein breakdown and free amino acid analyses to demonstrate how the differences in the structure of four isocaloric matrices (suspension, gel, foam, and heat-set foam) of a commercial potato protein ingredient impact its digestibility and therefore its protein quality [6]. A greater structural complexity was found to lead to more delayed gastric emptying. In addition to a slower gastric emptying rate, the heat-treated samples revealed a higher degree of hydrolysis and lower trypsin inhibitory activity than the non-heat-treated samples. Regarding the distribution of the bioaccessible peptides formed, similar trends were found between samples at different gastric and intestinal times, apart from the heat-set foam matrix. 

Following this line of research, the study of Jiménez-Barrios et al. (Contribution 4) characterized in detail the peptidome profile of duodenal digests from pigs as a recognized model of human digestion after oral administration of two dairy samples: micellar casein and a previously described casein hydrolysate [7]. When animals received micellar casein, a slower transit of nitrogen to the duodenum was observed, and the duodenal digests contained a wider range of peptide sizes as well as a higher number of sequences longer than five amino acids. Likewise, noticeable differences were revealed between the sequences identified in casein and hydrolysate digests, including the presence of different opioid peptides. Furthermore, the amino acid availability was quantified in plasma and evidenced a more rapid absorption in the hydrolysate group, with several branched-chain and other hydrophobic amino acids peaking at shorter times in comparison to the casein-fed animals. 

Specifically focused on food proteins (Contributions 1–4), these investigations represent not only a key tool to validate in vitro digestion protocols but also allow for the obtention of valuable data that could be applied to physiological and metabolic research studies [8,9]. The fate of food proteins during gastrointestinal digestion and the biological effects of their corresponding released peptides are thus highlighted as an area of intense research in the field of functional foods [10,11]. Hence, food-derived peptides with biological properties at the gastrointestinal level, such as antioxidant and antimicrobial [12], modulation of inflammation and immune response [13], chemoprevention of malignant diseases [14], opioid activity [15], or anti-diabetic effects [16], among others, have been reported in various investigations. 

Food researchers have also utilized gastrointestinal digestion protocols to evaluate the bioaccessibility and bioavailability of nutrients and bioactive compounds [17]. In this respect, the study of Fărcaș et al. (Contribution 5) used brewers’ spent grain, the main by-product obtained from the industrial brewing process, as a sustainable source of minerals and B group vitamins and evaluated its bioaccessibility. The study found that micro-minerals such as Fe, Cu, Zn, Cr, Mn, Ni, Ba, and Sr and macro-minerals such as Ca, Mg, Na, K, and B group vitamins (B1, B3, B6, and B12) presented different bioaccessibility values following the INFOGEST digestion methodology. Indeed, these authors suggested that the diverse sample types (including different percentages of malted cereals), their chemical composition (protein, fiber, bound polypeptide, and polysaccharide contents), along with the gastrointestinal digestion conditions, were the main factors that finally affected the bioaccessibility values of the assessed minerals and B vitamins. 

Regarding bioactive compounds, proanthocyanidins are a type of polyphenol with previously described preventive properties against obesity alterations [18]. In the study of Yu et al. (Contribution 6), persimmon proanthocyanidins significantly reduced the body weight of high-fat-diet-fed C57BL/6J mice, with different improvements in insulin resistance, lipid accumulation, as well as gut microbiota composition and diversity depending on their degrees of polymerization. Furthermore, in comparison with the oligomeric proanthocyanidins, polymeric proanthocyanidins exhibited strong inhibition over the activity of the digestive enzymes α-amylase and pancreatic lipase. In this line of research, previous studies had shown encouraging results for the inhibition of lipid digestion enzymes using bioactive compounds derived from black tea brew and grape seed powder [19,20]. Hence, the study of Tormási and Abrankó (Contribution 7) examined the lipolysis inhibitory effect of both ingredients when co-digested with two selected real foods with different lipid profiles as substrate tests (cream and baked beef). To that purpose, this study also applied the INFOGEST digestion protocol. The authors found that the characteristics of the dietary fat source can influence the lipolysis process, as both samples similarly decreased the lipolysis of the cream sample, but they were not able to influence the digestibility of beef fat, showing a simpler fatty acid profile in comparison with the diverse fatty acid composition of milk fat. Moreover, it was suggested that the lipolysis inhibitory effect of black tea brew and grape seed powder primarily affected the lipolysis of short- and medium-chain fatty acids because of the different preference of pancreatic lipase, in opposition to gastric lipase, to digest this type of triacylglycerol, which is further enhanced as a result of co-digestion with the evaluated ingredients.

Lastly, the Special Issue concludes with a review by Paterson et al. (Contribution 8) that summarizes the novel trends in microalgae research. This comprehensive review provides an updated discussion on the nutritional value (protein and amino acids, lipids and fatty acids, carbohydrates, minerals, vitamins, carotenoids, and phenolic compounds), biological effects (antioxidant, antimicrobial, and anticarcinogenic), as well as the digestibility of two microalgae genera, *Tetraselmis* and *Nannochloropsis*, as the basis to support their alternative exploitation as ingredients with health benefits and, consequently, with potential for the development of novel functional foods.
